# Clinical and laboratory characteristics of patients infected with *Shewanella* species at a tertiary hospital in Hefei City, China: a retrospective analysis

**DOI:** 10.3389/fcimb.2026.1700922

**Published:** 2026-02-02

**Authors:** Xiangyun Li, Xiaoqin Deng, Jun Xu, Boke Zhang, Xinyu Yan, Yuanhong Xu

**Affiliations:** 1 Department of Clinical Laboratory, The First Affiliated Hospital of Anhui Medical University, Hefei, Anhui, China; 2 Department of Emergency Medicine, The First Affiliated Hospital of Anhui Medical University, Hefei, Anhui, China; 3 Department of Clinical Laboratory, The First Affiliated Hospital of Anhui University of Chinese Medicine, Hefei, Anhui, China; 4 Department of Laboratory Medicine, Anhui Medical University, Hefei, Anhui, China

**Keywords:** clinical characteristics, hepatobiliary diseases and malignancy, laboratory characteristics, rare, *Shewanella* species (*Shewanella* spp.)

## Abstract

**Introduction:**

*Shewanella* species (*Shewanella* spp.) were emerging and rare pathogens. Very few studies had focused on *Shewanella* spp. infection due to its low incidence. A retrospective analysis summarizing clinical and laboratory characteristics of *Shewanella* spp. infection at a tertiary hospital in Hefei City was conducted to learn more about the rare bacterium.

**Methods:**

A total of 36 patients with *Shewanella* spp. infection from October 2019 to February 2025 were included. The data of all patients were collected by reviewing electronic records.

**Results:**

Among 36 isolated strains, 77.8% were *Shewanella algae* and 22.2% were *Shewanella putrefaciens*. Abdominal pain was the most common clinical symptom. Intrahepatic stone and cholangitis was the main diagnosed disease. According to the type of main diagnosed disease, they were divided into two groups: hepatobiliary disease group and non-hepatobiliary disease group. The laboratory results were analyzed, and it was revealed that the laboratory characteristics of anemia, neutrophilia, leukocytosis, and so on were common. Serum coagulation tests results showed that it was significantly higher than the normal value, and all other serum biochemical and coagulation tests results were mostly normal. For microorganism culture, co-infection microorganisms were obtained. *Shewanella* spp. were usually susceptible to aminoglycoside, quinolone, cephalosporin, carbapenems, and compound antibiotics. All patients were treated with antibiotics, and there were one or more types of antibiotics to use, all of whom had effective treatment outcomes.

**Discussion:**

*Shewanella* spp. infections were very limited. The study might improve the attention and awareness of the rare bacterial infection.

## Introduction

*Shewanella* species (*Shewanella* spp.) are gram-negative, rod-shaped, oxidase-positive, and facultative anaerobic and could produce hydrogen sulfide. It was first described by Derby and Hammer in 1931. They found and isolated an unknown bacterial strain from putrefied butter and water supplies of dairies and named the bacterium *Achromobacter putrefaciens.* In 1941, Long and Hammer renamed the new bacterium *Pseudomonas putrefaciens*, which was placed in *Pseudomonas* group in the next three decades and known as spoilage bacteria of fish. In 1974, it was classified as *incertae sedis* because the G + C content was below the content range of *Pseudomonas* spp., and in 1985, it was reclassified to the *Vibrionaceae* family and named *Shewanella putrefaciens* to commemorate James Shewan’s contribution to marine microbiology, including the related species *Shewanella hanedai* and *Shewanella benthica* (Yu et al., 2022; Holt et al., 2005). As mentioned above, *Shewanella putrefaciens* was the first species described in this genus, which caused food spoilage. Due to isolation from putrid butter, *Shewanella putrefaciens* as a food spoiler was deemed in several food products. In the 1990s, *Shewanella algae* was isolated according to genomic and phenotypic studies (Nozue et al., 1992)*. Shewanella algae* and *Shewanella putrefaciens* mainly differed in genomic sequence by 16S rRNA analysis, DNA–DNA hybridization, and other gene sequencing technologies.

*Shewanella* spp. are ubiquitously distributed in natural environments all over the world, and their main natural habitats are water and sediment. They could be found and isolated in marine environments, oil fields, and sediments. They play a great role in the turnover of organic material and are capable of dissimilatory reduction of various metals and other substances. Danish scientists isolated *Shewanella* spp. with a seawater salinity of 15%–20% and with the temperature of seawater at above 13°C during July to October in the 1990s (Yang et al., 2020; Petersen et al., 2018). They thought that the right salinity and temperature of the seawater were crucial to the survival. It is the reason that most *Shewanella* spp. infections occur in countries with a warm climate or where the summers are peak seasons.

Up to now, some species including *Shewanella xiamenensis* have continuously been detected (Abuladze et al., 2023; Zhang et al., 2023; García-Descalzo et al., 2022). Most *Shewanella* spp. detected in clinical specimens are *Shewanella algae* and *Shewanella putrefaciens*, and it seems that above 80% of isolates from human specimens are *Shewanella algae* (Vignier et al., 2013; Bernshteyn et al., 2020). *Shewanella* spp. are sometimes detected together with other bacteria (polymicrobic infection), and studies in animals have helped explore the pathogenicity (Huang et al., 2021; Mohr et al., 2016; Erfanmanesh et al., 2019). It had been reported that there were no pathogenic differences between *Shewanella algae* and *Shewanella putrefaciens*, but a pathogenic study in mice executed by Khashe and Janda came to the conclusion that *Shewanella algae* was more virulent, and the hemolytic activity was a key virulence factor (Khashe and Janda, 1998). In ear infections caused by *Shewanella putrefaciens*, researchers found that the ability of forming biofilms was also a pathogenic factor (Holt et al., 1997). *Shewanella* spp. has tolerance to bile salts and secretes extracellular siderophores and other exoenzymes, and it is reported that tetrodotoxin and pufferfish toxin could be produced (Li et al., 2023; Wang et al., 2020; Ghanei-Motlagh et al., 2019). Exposure to seawater, especially with a skin lesion or trauma, might be the main source of infection in humans and is reported many times in literatures (Chen et al., 2020; Yan et al., 2022). Other predisposing factors include the presence of hepatobiliary disease, malignancy, prematurity, and an impaired immune system (Liu et al., 2013; To et al., 2010; Liu et al., 2022). Community- and hospital-associated *Shewanella* spp. infections are not common (Zong, 2011). Fewer studies have focused on *Shewanella* spp. infection due to low incidence. Medline searches reveal that there are no reports in Hefei City. The clinical features of the patients are diverse and remain uncertain. Hence, we describe the clinical and laboratory characteristics of patients with *Shewanella* spp. infection in a tertiary hospital in Hefei City in China, hoping to provide more information and enhance the detection and diagnosis of *Shewanella* spp. infection.

## Materials and methods

### Recruitment criteria of *Shewanella* spp. infection

The retrospective study was performed at the First Affiliated Hospital of Anhui Medical University by including all patients infected with *Shewanella* spp. from October 2019 to February 2025. There was no established criteria of *Shewanella* spp. infection. Generally, a case was estimated to be positive when *Shewanella* spp. strain was isolated from the bile, bone marrow, blood, or cerebrospinal fluid (CSF) as identified by matrix-assisted laser desorption ionization time-of-flight mass spectrometry (MALDI-TOF MS, BioMérieux, France). *In vitro* antibiotic susceptibility testing was performed by automated VITEK 2 system and Kirby–Bauer disc agar diffusion methods, with interpretations based on CLSI M100 performance standards.

### Data collection

The data of all the enrolled patients were obtained by reviewing the electronic records. The demographic information was as follows: age, gender, basic diseases, hospitalization time, laboratory test results, types of antibiotics used, results of antibiotic susceptibility testing, and discharge status.

### Statistical analysis

Statistical analysis was carried out with SPSS 26.0. Data of continuous variables which assumed normal distribution were displayed as the mean ± standard deviation (SD) and compared by using Student’s *t*-test, and those without abnormal distribution were provided as medians (interquartile range (IQR)) and compared using Mann-Whitney *U-*test. For categorical variables, the data of categorical variables were analyzed as *n* (%) and compared by using Fisher’s exact test. *P*-values less than 0.05 and 0.001 were considered significantly different.

## Results

### Demographic and epidemiological characteristics

**Figure 1:**
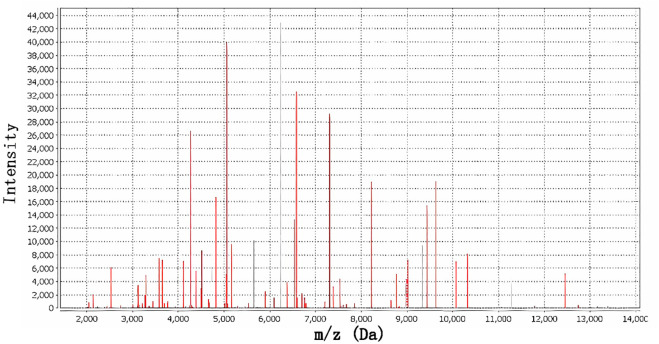
MALDI-TOF MS spectrum of *Shewanella algae* with 99.9% confidence.

**Figure 2:**
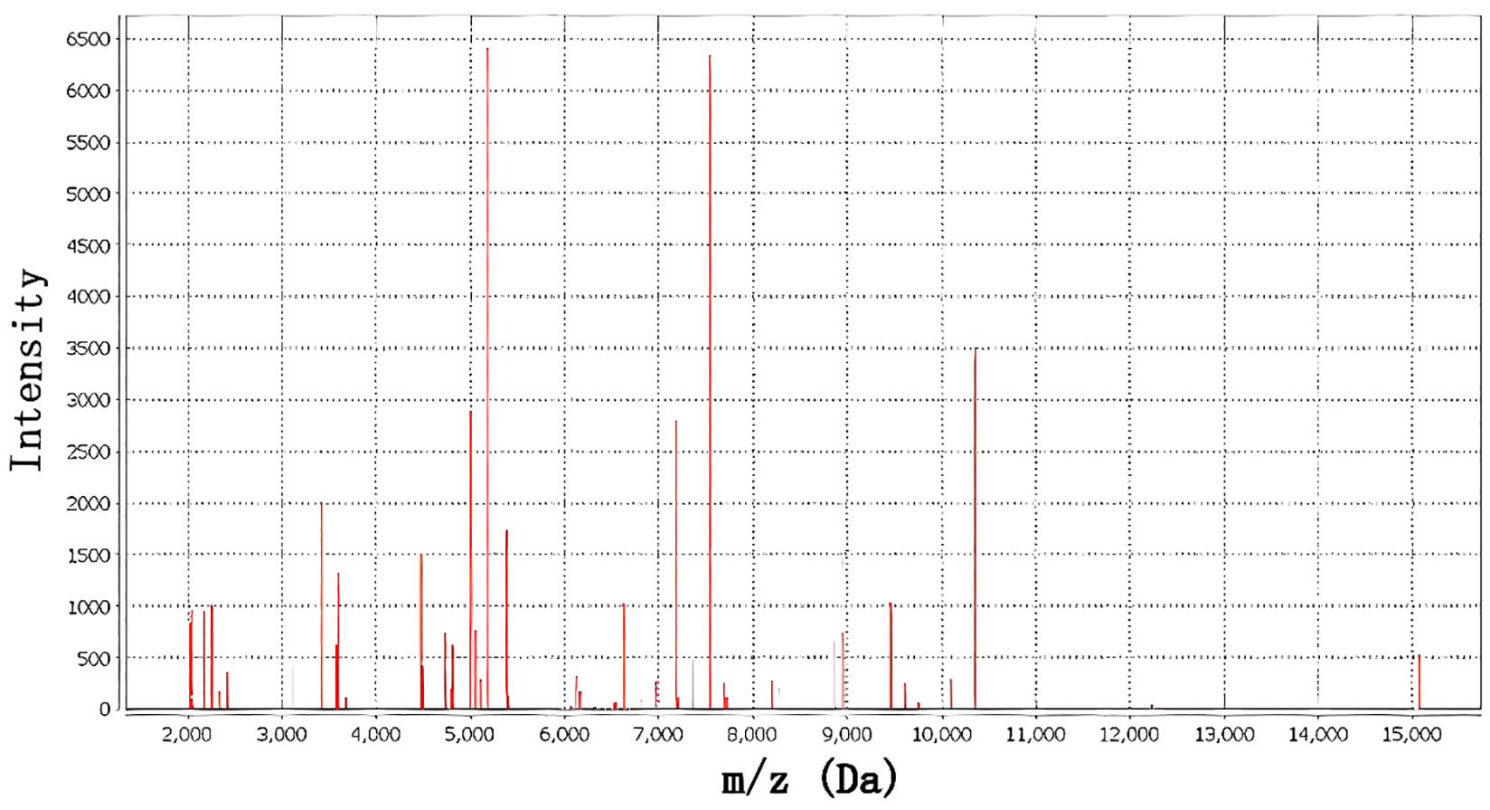
MALDI-TOF MS spectrum of *Shewanella putrefaciens* with 99.9% confidence.

Among 36 isolated strains, 77.8% were *Shewanella algae* and 22.2% were *Shewanella putrefacien* (**Figures 1**, **2**). The male and female patients were 61.1% and 38.9%, respectively, and the ratio of male to female was 1.57, without a significant difference. The range of age was from 3 to 94 years, and the average age was 58.44 years old. A total of 31 patients were over 45 years old, occupying the vast majority. There was not a significant difference in the number of positive patients in the four seasons of the year (*P* > 0.05). Intrahepatic stone and cholangitis were the primary reasons for patient admission (the main diagnosed disease). According to the type of primary reason, the patients were divided into two groups: hepatobiliary disease group (19 patients) and non-hepatobiliary disease group (17 patients). Cholecystectomy and hypertension were the most important underlying diseases, with eight and eight cases, respectively. The main positive patients were in the Department of Hepatopancreatobiliary Surgery and the Department of Infectious Diseases, accounting for 36.1% and 8.3%, respectively. The most common specimen types were bile and sputum, with a percentage of 33.3% and 22.2%, respectively. Compared to the non-hepatobiliary disease group, hepatocholangitis and stone-related disease as the basic diseases were more common in the hepatobiliary disease group (*P* < 0.001). The mean number of hospitalization days in the two groups were 21.4 ± 8.9 and 29.1 ± 23.9 days, respectively, and the difference was not significant (*P* > 0.05). 4 of the 36 patients were cured and discharged, and the other 32 patients were relieved and discharged with improved status, as listed in **Tables 1**, **2**.

**Table 1:** Detailed information of *Shewanella* spp.-positive patients in the hepatobiliary disease group (*n* = 19).

Patient number	Gender	Age	Department	Specimen type	Main diagnosis	Underlying illness	Discharge status	Group
1	M	33 years	Infectious diseases	Bile	Hepatocholangitis	Hepatitis B	Improved	H-B
2	F	51 years	Hepatopancreatobiliary surgery	Bile	Intrahepatic stone and cholangitis	Diabetes	Improved	H-B
3	F	57 years	Hepatopancreatobiliary surgery	Bile	Intrahepatic stone and cholangitis	Cholecystectomy	Improved	H-B
4	F	59 years	Hepatopancreatobiliary surgery	Bile	Space occupying lesion of bile duct	Cholecystectomy	Improved	H-B
5	F	64 years	Infectious diseases	Bile	Decompensated liver cirrhosis	Diabetes and biliary tract malignancy	Improved	H-B
6	M	69 years	Hepatopancreatobiliary surgery	Bile	Intrahepatic stone and cholangitis	Cholecystectomy	Improved	H-B
7	F	69 years	Hepatopancreatobiliary surgery	Bile	Choledocholithiasis	Cholecystectomy	Cured	H-B
8	F	50 years	Emergency ICU	Bile	Intrahepatic stone and cholangitis	Cholecystectomy	Improved	H-B
9	M	85 years	Hepatopancreatobiliary surgery	Bile	Obstructive jaundice (pancreatic head carcinoma) and Biliary tract infection	Hypertension	Improved	H-B
10	F	50 years	Gastroenterology	Secreta	Bile duct malignancy	Intrahepatic bile duct and common bile duct lithotomy	Improved	H-B
11	F	50 years	Infectious diseases	Blood	Decompensated liver cirrhosis	Cholecystectomy	Improved	H-B
12	M	64 years	Hepatopancreatobiliary surgery	Drainage liquid	Bile duct malignancy	Hypertension	Improved	H-B
13	M	67 years	Hepatopancreatobiliary surgery	Bile	Choledocholithiasis with acute cholecystitis and hepatic cysts	Fracture of collarbone and patella	Cured	H-B
14	F	54 years	Hepatobiliary and pancreatic transplantation surgery	Puncture fluid	Obstructive jaundice	Cholecystectomy	Cured	H-B
15	M	68 years	Hepatobiliary and pancreatic transplantation surgery	Bile	Intrahepatic bile duct calculus with cholangitis	Operation of biliary tract	Improved	H-B
16	M	64 years	Hepatopancreatobiliary surgery	Blood	Hepatolithiasis with cholangitis, liver abscess, pancreatic surgery	Duodenectomy	Improved	H-B
17	M	71 years	Gastroenterology	Puncture fluid	Choledocholithiasis with cholangitis	Hypertension	Improved	H-B
18	M	55 years	Hepatobiliary and pancreatic transplantation surgery	Puncture fluid	Hepatolithiasis	Cholecystectomy	Improved	H-B
19	M	70 years	General medicine	Bile	Obstructive jaundice	Pancreatic cancer	Improved	H-B

F, female; M, male; H-B, hepatobiliary disease; Non-H-B, non-hepatobiliary disease.

**Table 2:** Demographics and clinical characteristics of patients with *Shewanella* spp. infection (*n* = 36).

Variable	Total (*n* = 36)	Hepatobiliary disease group (*n* = 19)	Non-hepatobiliary disease group (*n* = 17)	*P*-value
Age, years	58.4 ± 16.6	61.7 ± 11.2	54.8 ± 20.8	0.219
0–45 years	14 (38.9)	1 (7.1)	13 (92.9)	
>45 years	22 (61.1)	18 (81.8)	4 (18.2)	<0.001^**^
Gender				
Male	22 (61.1)	10 (52.6)	12 (70.6)	
Female	14 (38.9)	9 (47.4)	5 (29.4)	0.151
Diagnosis season				
Spring (1–3)	9 (25.0)	5 (26.3)	4 (23.5)	
Summer (4–6)	8 (22.2)	4 (21.1)	4 (23.5)	
Autumn (7–9)	9 (25.0)	5 (26.3)	4 (23.5)	
Winter (10–12)	10 (27.8)	5 (26.3)	5 (29.4)	0.528
Mean hospitalization days	25.0 ± 17.8	21.4 ± 8.9	29.1 ± 23.9	0.207
Basic disease				
Hepatocholangitis	10 (27.8)	10 (52.6)	0 (0.0)	<0.001^**^
Stone-related disease	13 (36.1)	13 (68.4)	0 (0.0)	<0.001^**^
Malignancy	8 (22.2)	6 (31.6)	2 (11.8)	0.122
Decompensated liver cirrhosis	2 (5.6)	2 (10.5)	0 (0.0)	0.271
End-stage renal disease	2 (5.6)	0 (0.0)	2 (11.8)	<0.001^**^
Cardiovascular disease	4 (11.1)	0 (0.0)	4 (23.5)	<0.001^**^

Data are expressed as percent (%) or means ± standard deviations (SD). H-B versus non-H-B.

**P <0.001 were considered statistically significant.

### Clinical characteristics

The most common clinical symptom was abdominal pain (50.0%), followed by fever (11.1%), icterus (11.1%), abdominal distension (8.3%), and vomiting (8.3%). Abdominal pain was observed more in the hepatobiliary disease group (*P* < 0.001). The mean body temperature was 37.13°C ± 1.18°C in all patients, and it had no significant differences (*P* > 0.05, **Table 3**). In terms of complications, electrolyte disorder occurred in 17 patients (47.2%), followed by pneumonia (13.9%). The complication of pneumonia was more likely to appear in the non-hepatobiliary disease group, and it had significant differences (*P* < 0.05).

**Table 3:** Clinical symptoms analysis of patients with *Shewanella* spp. infection (*n* = 36).

Clinical symptoms	Total *n* = 36 (%)	Hepatobiliary disease group *n* = 19 (%)	Non-hepatobiliary disease group *n* = 17 (%)	*P*-value
Abdominal pain	18 (50.0)	15 (79.0)	3 (17.7)	<0.001^**^
Abdominal distension	3 (8.3)	2 (10.5)	1 (5.9)	0.407
Diarrhea	2 (5.6)	0 (0.0)	2 (11.8)	0.216
Vomit	3 (8.3)	1 (5.3)	2 (11.8)	0.361
Icterus	4 (11.1)	4 (21.1)	0 (0.0)	0.066
Fever	4 (11.1)	3 (15.8)	1 (5.9)	0.280
Mean body temperature(°C)	37.1 ± 1.2	37.0 ± 1.2	36.7 ± 0.7	0.334
Melena	1 (2.8)	1 (5.3)	0 (0.0)	0.528
Complication				
Electrolyte disorder	17 (47.2)	11 (57.9)	6 (35.3)	0.109
Pneumonia	5 (13.9)	0 (0.0)	5 (29.1)	0.016^*^

Data are expressed as percent (%) or means ± standard deviations (SD). H-B versus non-H-B. *P <0.05 and **P <0.001 were considered statistically significant.

### Laboratory characteristics

The laboratory results of blood routine, biochemical, and coagulation tests were analyzed and revealed the laboratory characteristics of neutrophilia (26 cases, 72.2%), leukocytosis (24 cases, 66.8%), anemia (27 cases, 75.0%), hypoproteinemia (17 cases, 47.2%), hyperbilirubinemia (18 cases, 50.0%) and hepatic injury (25 cases, 69.4%) that were more common in all patients, as listed in **Table 4**. Serum coagulation tests showed elevated PT levels, and the mean value of PT was 14.37 ± 1.73 s, which was significantly higher than the normal value (9.0–13.0 s). All other serum biochemical and coagulation testing results were mostly within the normal range. Hepatic injury occurred in 18 (94.7%) patients in the hepatobiliary disease group as well as in seven (4.1%) patients in the non-hepatobiliary disease group, and the statistical analysis showed that hepatic injury was more frequent in the former group (*P* < 0.001). The objective and reliable biochemical indicators of hepatic injury such as glutamic–pyruvic transaminase (ALT), glutamic oxalacetic transaminase (AST), alkaline phosphatase (ALP), and γ-glutamyl transpeptidase (GGT) all had significant differences between the two groups (all *P* < 0.05). Besides that, hyperbilirubinemia occurred in 15 (78.6%) cases and three (17.7%) cases, respectively, and the statistical analysis showed that hyperbilirubinemia was significantly different (*P* < 0.05). Frequently used indicators of hyperbilirubinemia such as total bilirubin (TBIL), direct bilirubin (DBIL) and indirect bilirubin (IBIL) showed significant differences (all *P* < 0.05). Indicators of hypoproteinemia such as albumin (ALB) and A/G displayed significant differences (all *P* < 0.05). Serum uric acid (UA) was significantly different (*P* < 0.05), and the level was lower in the hepatobiliary disease group than those in the non-hepatobiliary disease group. The WBC, NEUT, and PLT counts were significantly different (all *P* < 0.05), and the other testing data were not significantly different.

**Table 4:** Laboratory results of patients with *Shewanella* spp. infection (*n* = 36).

Laboratory results	Total n = 36 (%)	Hepatobiliary disease group n = 19 (%)	Non-hepatobiliary disease group n = 17, (%)	P-value
Hepatic injury^a^	25 (69.4)	18 (94.7)	7 (4.1)	< 0.001**
ALT	54.9 (28.2–126.4)	81.8 (36.3–159.1)	24.9 (13.5–40.6)	0.041*
AST	76.9 (35.6–147.0)	116.0 (53.4–185.3)	33.1 (12.0–71.6)	0.043^*^
ALP	234.7 (86.1–329.3)	353.9 (113.3–642.0)	101.6 (51.4–112.3)	0.046^*^
GGT	175.6 (13.2–256.4)	254.9 (15.3–347.4)	129.7 (11.1–134.8)	0.027^*^
Hyperbilirubinemia^b^	18 (50.0)	15 (78.9)	3 (17.7)	< 0.001**
TBIL	60.3 (9.2–104.1)	102.7 (14.5–176.3)	12.9 (6.2–21.3)	0.032*
DBIL	50.2 (28.3–95.3)	82.1 (49.2–102.2)	3.6 (2.2–17.1)	0.012*
IBIL	19.1 (6.6–36.6)	22.9 (9.2-55.2)	9.5 (3.1–24.2)	0.039*
Hypoproteinemia^c^	17 (47.2)	11(57.9)	6 (35.3)	0.109
TP	61.9 (48.3–79.1)	63.3 (54.2–76.9)	60.7 (46.5–82.0)	0.367
ALB	35.8 ± 5.7	38.3 ± 5.5	33.58 ± 5.0	0.009*
GLO	26.2 (16.4–35.8)	24.9 (18.3–39.7)	27.3 (15.7–34.2)	0.267
A/G	1.5 (0.76–2.17)	1.3 (0.55–1.96)	1.6 (0.99–2.26)	0.048*
UA	253.9 (79.2–302.7)	187.8 (66.1–235.1)	327.9 (123.2–426.7)	0.031*
Cr	161.5 (157.2–396.7)	50.7 (326.2–393.4)	221.8 (116.9–408.3)	0.616
GLU	6.9 (4.8–17.2)	6.8 (4.4–13.7)	7.0 (5.6–18.5)	0.936
K	4.0 (3.8–6.3)	3.9 (3.6–5.1)	4.2 (4.1–6.5)	0.521
Na	138.4 (133.8–142.3)	137.2 (131.2–139.7)	139.6 (134.2–144.5)	0.358
Leukocytosis^d^ (>9.5 × 10^9^)	24 (66.7)	16 (84.2)	8 (47.1)	0.188
WBC counts (×10^9^)	11.5 (7.3–14.6)	13.7 (7.6–17.8)	9.0 (6.8–13.1)	0.032*
Neutrophilia^e^ (> 6.3 × 10^9^)	26 (72.2)	17 (89.5)	9 (52.4)	0.016*
NEUT# (×10^9^/L)	9.9 (5.7–11.5)	12.3 (5.8–12.6)	7.4 (5.1–10.8)	0.023*
LYMPH# (×10^9^/L)	0.7 (0.5–1.6)	0.8 (0.6–1.8)	0.6 (0.4–1.5)	0.425
MONO# (×10^9^/L)	0.5 (0.5–0.8)	0.6 (0.5–0.8)	0.5 (0.4–0.7)	0.782
Anemia^f^	27 (75.0)	13 (68.4)	14 (82.4)	0.196
Hb (g/L)	106.8 (83.3–122.4)	105.7 (78.2–119.5)	108.6 (88.1–128.6)	0.729
RBC counts (×10^12^/L)	3.6 (2.6–4.2)	3.5 (2.5–4.1)	3.6 (2.7–4.3)	0.613
HCT (%)	32.5 (28.5–39.2)	32.2 (26.2–38.5)	32.9 (29.5–40.1)	0.802
PLT counts (×10^9^/L)	174.7 ± 82.08	147.8 ± 72.62	204.8 ± 83.55	0.035*
APTT(s)	35.3 (33.6–42.7)	34.1 (32.6–41.6)	36.9 (35.1–43.5)	0.527
PT(s)	28.4 (23.6–45.6)	25.3 (19.6–42.7)	32.4 (26.8–49.1)	0.642
INR	1.1 (1.0–1.2)	1.1 (0.9–1.2)	1.1 (1.0–1.3)	0.921
TT (s)	17.1 (15.7–17.9)	17.3 (15.9–18.1)	17.0 (15.5–17.6)	0.425
FIB (g/L)	3.9 (3.6–5.5)	3.9 (3.7–5.6)	3.8 (3.5–4.3)	0.836

Data are expressed as percent (%) or means ± standard deviations (SD). H-B versus non-H-B. *P <0.05 and **P <0.001 were considered statistically significant.

ALT, aspartate aminotransferase; AST, aspartate aminotransferase; ALP, alkaline phosphatase; GGT, γ-glutamyl transpeptidase; TP, total protein; ALB, albumin; GLO, globulin; A/G, albumin/globulin ratio; TBIL, total bilirubin; UA, uric acid; Hb, hemoglobin; RBC, red blood cell; HCT, hematocrit; WBC, white blood cell; NEUT#, neutrophil counts; LYMPH#, lymphocyte counts; MONO#, monocyte counts; PLT, platelet; PCT, procalcitonin; APTT, activated partial thromboplastin time; PT, prothrombin time; INR, international normalized ratio; TT, thrombin time; FIB, fibrinogen.

a Hepatic injury: ALT, male >50 U/L, female >40 U/L; AST, male >40 U/L, female >35 U/L; ALP, male >125 U/L, female >100 U/L; GGT, male >60 U/L, female >45 U/L.

b Hyperbilirubinemia: TBIL >23.0 µmol/L; DBIL >8.0 µmol/L, or IBIL>15.0 µmol/L.

c Hypoproteinemia: TP <60 g/L or ALB <35 g/L.

d Leukocytosis: WBC counts >9.5 × 10^9^/L.

e Neutrophilia: Neutrophil counts >6.3 × 10^9^/L.

f Anemia: male Hb <130 g/L and female Hb <115 g/L.

### Co-infection organisms

Among 36 patients with *Shewanella* spp. infection, 36 specimens were available for microorganism culture, and co-infection microorganisms were obtained (28 cases, 77.8%). *Escherichia coli* (8 cases, 22.2%), *Klebsiella pneumoniae* (5 cases, 13.9%), and *Enterococcus faecium* (3 cases, 8.3%) were the top three. Other gram-negative and gram-positive bacteria and fungi were also detected, and the results are listed in **Table 5**.

**Table 5:** Co-infection microorganisms in patients with *Shewanella* spp. infection (*n* = 36).

Co-infection organisms	Total *n* = 36 (%)	Hepatobiliary disease group *n* = 19 (%)	Non-hepatobiliary disease group *n* = 17 (%)	*P*-value
No co-infection	8 (22.2)	4 (21.1)	4 (23.5)	0.304
Co-infection(main)	28 (77.8)	15 (79.0)	13 (76.5)	0.305
*Escherichia coli*	8 (22.2)	7 (36.8)	1 (5.9)	0.028*
*Klebsiella pneumoniae*	5 (13.9)	1 (5.3)	4 (23.5)	0.119
*Acinetobacter baumannii*	1 (2.8)	0 (0.0)	1 (5.9)	0.472
*Enterococcus faecium*	3 (8.3)	3 (15.8)	0 (0.0)	0.136
*Enterococcus faecalis*	1 (2.8)	1 (5.3)	0 (0.0)	0.528
*Enterococcus gallinarum*	1 (2.8)	1 (5.3)	0 (0.0)	0.528
*Staphylococcus haemolyticus*	1 ((2.8)	0 (0.0)	1 (5.9)	0.472
*Candida albicans*	2 (5.6)	1 (5.3)	1 (5.9)	0.513

Data are expressed as percent (%) or means ± standard deviations (SD). H-B versus non-H-B. *P <0.05 was considered statistically significant.

### Antibiotic susceptibility testing, antibiotic treatment history, and outcomes

Aminoglycoside antibiotics (amikacin and tobramycin), quinolone antibiotics (ciprofloxacin and levofloxacin), cephalosporin antibiotics (ceftazidime and ceftriaxone), carbapenems antibiotics (meropenem and imipenem), and compound antibiotics (cefperazone–sulbactam and piperacillin–sulbactam) were selected for antibiotic susceptibility testing, and most of them were commonly used as clinical antibiotic. The results of the antibiotic susceptibility of *Shewanella* spp. are shown in **Table 6**. The identified bacterial strains were usually susceptible to tobramycin (24 cases, 66.7%), ciprofloxacin (24 cases, 66.7%), levofloxacin (23 cases, 63.9%), ceftazidime (25 cases, 69.4%), ceftriaxone (25 cases, 69.4%), meropenem (25 cases, 69.4%), imipenem (25 cases, 69.4%), cefperazone–sulbactam (26 cases, 72.2%), and piperacillin–sulbactam (26 cases, 72.2%). All testing data were not significantly different between the two groups. All 28 patients were treated with antibiotics (100.0%), and one or more types of antibiotics were used. The mean number of types of antibiotics was 1.47 ± 0.96 in all patients, and it was not significantly different between the two groups. After treatment, all 36 patients (100.0%) had effective treatment outcomes, and no patient died during hospitalization.

**Table 6:** Antibiotic susceptibility, antibiotic treatment history, and outcomes of patients with *Shewanella* spp. infection (*n* = 36).

	Total *n* = 36 (%)	Hepatobiliary disease group *n* = 19 (%)	Non-hepatobiliary disease group *n* = 17 (%)	*P*-value
Antibiotic susceptibility testing	Susceptive	Susceptive	Susceptive	
Amikacin	24 (66.7)	13 (68.4)	11 (64.7)	0.268
Tobramycin	24 (66.7)	13 (68.4)	11 (64.7)	0.268
Ciprofloxacin	24 (66.7)	13 (68.4)	11 (64.7)	0.268
Levofloxacin	23 (63.9)	12 (63.2)	11 (64.7)	0.269
Ceftazidime	25 (69.4)	14 (73.7)	11 (64.7)	0.240
Ceftriaxone	25 (69.4)	14 (73.7)	11 (64.7)	0.240
Meropenem	25 (69.4	14 (73.7)	11 (64.7)	0.240
Imipenem	25 (69.4)	14 (73.7)	11 (64.7)	0.240
Cefperazone–sulbactam	26 (72.2)	14 (73.7)	12 (70.6)	0.283
Piperacillin–sulbactam	26 (72.2)	14 (73.7)	12 (70.6)	0.283
Antibiotic susceptibility history				
Antibiotic treatment	28 (100.0)	19 (100.0)	17 (100.0)	1.000
Mean number of antibiotics	1.47 ± 0.96	1.28 ± 0.79	1.68 ± 1.32	0.316
Outcomes				
Effective treatment for infection	28 (100.0)	19 (100.0)	17 (100.0)	1.000
In-hospital mortality	0 (0.0)	0 (0.0)	0 (0.0)	1.000

Data are expressed as percent (%) or means ± standard deviations (SD). H-B versus non-H-B.

## Discussion

There were more than 70 *Shewanella* species, and the main pathogenic species to human were *Shewanella algae* and *Shewanella putrefaciens* (Mukherjee et al., 2020). Most of the *Shewanella* spp. infections were opportunistic infections, and the related infectious diseases mainly included hepatobiliary system infection, skin and soft tissue infection, septicemia, otitis media, myelitis, and peritonitis. *Shewanella* spp. infections were gradually being recognized in China and had been reported as a pathogen in Beijing City and Shandong Province (Dai et al., 2019; Cao et al., 2021). Due to the rarity and low incidence, there were no reports in Hefei City in Anhui Province, which was located in the southeastern part of China. In our study, clinical and laboratory characteristics were described and analyzed in the First Affiliated Hospital of Anhui Medical University in the past 6 years (Shi et al., 2021a; Shi et al., 2021b). To our knowledge, this might be the first study which analyzed the data of patients with *Shewanella* spp. infection in Hefei City. In the study, *Shewanella algae* was more than *Shewanella putrefaciens* (28 vs. 8), which was similar to those in many provinces or cities in China and abroad. The patients aged 45 and above accounted for the majority, which proved that older age groups were the susceptible population. In the study of Ng et al., 61.7% were male patients, and the average age was 78 years in all 128 patients (Ng et al., 2022). Yu et al. reported that the age of people with *Shewanella* spp. infections ranged from neonates to 92 years old, and the elderly accounted for the largest proportion (34.43%) in comparison with the other age groups (Yu et al., 2022). All 36 cases were sporadic infections, and we concluded that it was not an outbreak. The causes of *Shewanella* spp. infections were always complex and could be influenced by some risk factors. Generally speaking, there were three predisposing factors. Firstly, exposure to marine environment was the most common, including occupational and recreation exposure, undercooked seafood exposure, and wound exposure to marine organisms. A number of patients became infected after exposure to soil, poultry, dairy products, or freshwater fish. Secondly, *Shewanella* spp. infections usually occurred in immunocompromised patients, including those with hepatobiliary disease, malignancies, and so on. Some studies had reported that hepatic disease might be a strong risk factor. The reason might be that the liver was a large immune organ in the body which contained more macrophages, Kupffer cells, lymphocytes, and so on. These cells had powerful immune functions and could eliminate bacteria and viruses from the body. In our study, the liver function was greatly impaired when patients have liver disease. It would lead to decreased immunity, and the resistance to *Shewanella* spp. infection becomes weak. *Shewanella* spp. would grow in abundance and cause infection. The immunity of the liver was unable to clear a large number of pathogenic bacteria, and liver function continues to be damaged. Eventually, decompensated liver cirrhosis occurs. End-stage renal disease referred to the end stage of various chronic kidney diseases. When patients had low immunity or immune disorders, there is invasion of viruses and bacteria (including *Shewanella* spp.) and some other disease-causing factors into the body. Due to the low immunity or immunity disorder, these pathogenic factors and antibodies were overimmunized, and it would produce a large number of immune complexes. These immune complexes were continuously deposited in the kidney, which led to the damage of the inherent kidney cells. The nephritis worsened, and eventually end-stage renal disease occurred. Thirdly, nosocomial infection was also a risk factor which led to the outbreaks of healthcare-associated *Shewanella* spp. infections. The source of infection was invasive procedures (intubation and catheterization). Oh et al. reported that the outbreak of *Shewanella algae* and *Shewanella putrefaciens* infections was caused by a shared measuring cup in a general surgery unit in Korea (Oh et al., 2008). In our study, hepatolithiasis and cholangitis were the main diagnostic diseases, and bile was the most abundant specimen type. Most infections occurred in the departments of Hepatobiliary Surgery and Infectious Diseases. It was related to the abnormal anatomical structure of the hepatobiliary system, decreased immune function, intestinal barrier dysfunction in patients with hepatobiliary diseases, and the biological characteristics of *Shewanella* spp. with salt tolerance and lipolysis, which could allow them to survive in the biliary system.

The most common clinical symptom was abdominal pain, followed by fever, abdominal distension, vomiting, and icterus. Abdominal pain was observed more in the hepatobiliary disease group. These symptoms were consistent with the clinical manifestations of hepatobiliary diseases. Janda et al. reported that *Shewanella*-related syndromes could be divided into five categories. One of them was hepatobiliary diseases and the other four were invasive diseases, skin and soft tissue infections, otitis media, associated sequela, and other infections (Janda and Abbott, 2014). *Shewanella* spp. infections were relatively weak virulent, and few people had a high fever. The complications caused by infection were summarized, and it was found that the level of serum sodium were low. The reason might be that serum sodium was lost too much through the gastrointestinal tract, such as by vomiting and gastrointestinal and biliary drainage, which could lead to excessive electrolyte loss. Pneumonia was another relatively common complication which was mainly concentrated in the non-hepatobiliary diseases group, and there was a significant difference. The incidence of pneumonia was close to 20%, and pneumonia was usually caused by upper respiratory infections. A total of four patients had the complication; one of them was admitted for respiratory failure due to sewage inhalation, and the other three patients were hospitalized for more than 1 month. We conclude that the upper respiratory infection was due to impaired immune function.

In the infection-related diseases, neutrophils and WBC counts in peripheral blood were significantly elevated (47.2% and 41.7%), while lymphocyte counts decreased and monocyte counts did not change significantly. We guessed that the observed differences in WBC, NEUT, and PLT counts in the two groups were attributable for the hepatobiliary disease. The increases of WBC, NEUT, and PLT counts were not influenced solely by a single factor as other contributing factors cannot be excluded. The results of hemoglobin, red blood cell, and hematocrit counts also decreased significantly. Correspondingly, the incidence of neutrophilia (47.2%), leukocytosis (41.7%), and anemia (33.3%) occurred in the majority of *Shewanella* spp. infection cases. As hepatobiliary disease was used as the basis of grouping, laboratory testing of hepatic injury and hyperbilirubinemia was performed, and the results of ALT, AST, ALP, and GGT (hepatic injury markers) as well as TBIL, DBIL, and IBIL (hyperbilirubinemia markers) increased obviously and had significant differences. When the liver suffers from an injury, albumin would decrease because the liver is the organ that produces albumin. Meanwhile, globulin would increase, and it might be part of the normal defenses against invading bacteria in diseases due to infections. Therefore, TP, Alb, Glo, and A/G (hypoproteinemia markers) had corresponding changes. In our study, the level of serum uric acid was low in the hepatobiliary diseases group, and it had a significant difference between the two groups. The reason might be that the liver contributed to the synthesis of uric acid and hepatobiliary disease often led to the occurrence of hypouricemia. There were no obvious changes in glucose, potassium, creatinine, and coagulation function. Co-infection microorganisms were frequently isolated in *Shewanella* spp. infections. It might be caused by immune deficiency. Interpretive breakpoints of minimum inhibitory concentrations (MICs) for *Shewanella* spp. had not been stipulated by CLSI until now. Huang et al. interpreted the breakpoints as susceptible, intermediate, and resistant in accordance with the criteria for the interpretation of *Enterobacteriaceae* to determine the results of antibiotic susceptibility testing for *Shewanella* spp. infection (Huang et al., 2022; Yousfi et al., 2017). We chose to adopt Huang’s experimental method and interpretation standard. The results showed that *Shewanella* spp. were mostly susceptible to many antibiotics, which was consistent with the results of previous studies. All of the infected patients were treated with antibiotics, and many patients were treated with more than one antibiotic. Effective treatments for infections were obtained.

## Conclusions

In conclusion, the most common clinical symptom in patients with *Shewanella* spp. infection was abdominal pain. Hepatobiliary disease or malignancy and an impaired immune system as underlying diseases were risk factors for *Shewanella* spp. infection. Most of the patients had distinct laboratory characteristics, including neutrophilia, leukocytosis, and anemia as infection indexes and hyperbilirubinemia and hypoproteinemia as hepatic injury indexes. The study summarized the data of patients at a tertiary hospital in Hefei City in China in the last 6 years, and it might provide an epidemiological and microbiological understanding of *Shewanella* spp. infection and some reference values for the treatments.

## Data Availability

The raw data supporting the conclusions of this article will be made available by the authors, without undue reservation.
